# Heavy metal and microbial safety assessment of raw and cooked pumpkin and *Amaranthus viridis* leaves grown in Abakaliki, Nigeria

**DOI:** 10.1002/fsn3.739

**Published:** 2018-08-06

**Authors:** Fidelis Azi, Micheal O. Odo, Peter A. Okorie, Helen A. Njoku, Veronica N. Nwobasi, Esther David, Theresa C. Onu

**Affiliations:** ^1^ Department of Food Science Ebonyi State University EBSU Abakaliki Nigeria; ^2^ Department of Home Economics Enugu State College of Education (Technical) Enugu Nigeria

**Keywords:** 16S rDNA, *Amaranthus viridis*, heavy metals, ITS rDNA, microbial safety, pumpkin

## Abstract

In this study, heavy metal and microbial safety assessment of raw and cooked pumpkin and *Amaranthus viridis* leaves grown in Abakaliki, Nigeria, was examined. The levels of lead (Pb), arsenic (As), chromium (Cr), cadmium (Cd), and mercury (Hg) were evaluated using atomic absorption spectrophotometer. The microbial cells were counted and further identified to species level using 16S rDNA and ITS rDNA sequencing analysis at CABI microbial identification services United Kingdom (UK). The results showed that the heavy metal concentrations of lead (10.5, 12.0), arsenic (7.5, 8.5), chromium (0.9, 0.1), and mercury (13.1, 14.0) in the pumpkin and *A. viridis* leaves, respectively, were above maximum acceptable limit according to relevant national and international food regulatory agencies (Tables 1 and 2). Cooking significantlyreduced the concentrations of the heavy metals at (*p* > 0.05) to or below, lead (6.8, 8.4), arsenic (5.1, 6.1), chromium (0.6.0.1), and mercury (9.5, 11.4) in the pumpkin and *A. viridis* leaves, respectively, but still not to international safe limit. The result of the microbial safety assessment showed that the microbial load of both the pumpkin and *Amaranthus viridis* leaves were above acceptable limit and the contaminating organisms were identified as *Escherichia coli* (504743), *Klebsiella pneumonia* (504744b), and *Aspergillus flavus* (504740).This study therefore shows that the vegetables (pumpkin and *A. viridis* leaves) contain unacceptable levels of toxic heavy metals and potentially dangerous pathogenic microorganisms, thus present significant health risk for the consumers.

## 1 INTRODUCTION

Vegetables constitute an essential part of human diet and are considered to play an important role in a healthy diet, providing important vitamins, minerals, antioxidant, and phytochemicals. Vegetables are grown in a natural environment and are therefore vulnerable to contamination with heavy metals and pathogens from different sources, including soil, irrigation, and postharvest water (Buck, Walcott, & Beuchat, 2003; Anon, 2005). These vegetables take up the metals by absorbing them from contaminated soil, water, as well as from deposits on different parts of the vegetables exposed to the air from polluted environments to high concentrations thereby causing serious health risk for the consumers (Sobukola, Adeniran, Odedairo, & Kajihausa, 2010). Thus, vegetables often contain both essential and toxic elements over a wide range of concentrations depending on environment of cultivation (Choi, 2011; EC 2004).

Vegetables are often consumed fresh, processed or semiprocessed; hence, consumers’ demand for better quality vegetables is increasing (Itanna, 2003). The perceptions of what is regarded as a better quality vegetable are somewhat subjective. Some consumers consider undamaged dark green and big leaves as characteristics of quality leafy vegetables. However, many consumers of vegetables are more critical of not just the external morphology but its safety from potentially pathogenic enteric microbes and toxic elements (Anon, 2005). Hence there is need for continuous scientific assessment of the heavy metal and microbial safety of leafy vegetables especially those grown in urban areas with deposit of industrial and domestic wastes (Burnett & Beuchat, 2001; CDC, 2006; EFSA, 2004). Heavy metals rank high among the major contaminants of leafy vegetables (Mapanda, Mangwayana, Nyamangara, & Giller, 2005; JECFA, 2003). Although certain heavy metals (chromium [Cr], manganese [Mn], nickel [Ni], copper [Cu], and iron [Fe]) are essential components for various biological activities within the human body, elevated levels of them can cause numerous health consequences to mankind. This is because heavy metals have the ability to accumulate in the leaving organisms (bioaccumulation) and at elevated level can be toxic (EFSA, 2009). For instance, when lead exceeds its safe value concentration, it causes hepatic and kidney damage, hemolytic anemia and methanoglobinemia. The acceptable limit for human consumption of lead for all food in solid form is 6 ppm, while for foods in liquid form, it is 1 ppm (FSAI, 2009; EC, 2004). Cadmium exerts its toxic effects on human health when present at higher concentration and causes severe diseases such as tubular growth, excessive salivation, gastrointestinal irritation, cancer, kidney damage, diarrhea, and vomiting (EFSA, 2009; EC, 2008). Report of heavy metal contamination of leafy vegetables has continued to be on the increased globally especially among vegetables grown in urban areas (Wilson, 2011). This is because increased urbanization and leafy vegetables are now grown in soil with high deposit of industrial and domestic wastes (Muchuweti et al., 2005). Thus, heavy metals such as cadmium, chromium, lead, arsenic, copper, and zinc have all been obtained in vegetables planted within urban areas and in most times in more than allowable limits (FSANZ, 2011; Itanna, 2003). Knowledge of metal–plant interactions is important for the safety of the environment and for reducing the risk associated with the introduction of trace metals into the food chain. Consequently, the metal can inactivate many important enzymes resulting in inhabitation of photosynthesis respiratory rate and other metabolic process in plant (Yebpella et al., 2011).

Microbial contamination of fresh vegetables on the farm and/or during handling is a common problem globally, and consumption of such vegetables has been established to be one of the leading causes of food poisoning worldwide (CDC, 2006; Anon, 2005). According to FAO and WHO convened Expert Meeting of the Codex Committee on Food Hygiene on the microbiological hazards associated with fresh produce; Leafy green vegetables were recommended to be given the highest priority in terms of fresh produce safety from a global perspective (CAC, 2003). Thus, it is now commonly accepted that fruit and leafy vegetables consumption is a risk factor for infection with enteric pathogens. So many enteric pathogens have been isolated from vegetables grown in a soil/irrigated with water contaminated with industrial and domestic sewage. Salad vegetables such as carrots, radishes, tomatoes, lettuce, cabbage, cucumbers, coriander have all been contaminated with *Escherichia coli*,* Enterobacter* spp., *Klebsiella* spp., *Salmonella typhi*,* Serratia* spp., *Providencia* spp., *Staphylococcus aureus* and *Pseudomonas aeruginosa* and other potentially pathogenic microbes (Francis, Thomas, & O'Beirne, 1999; Burnett & Beuchat, 2001). Recent examples of outbreaks related to fresh vegetables include cases of *E. coli* O157:H7, *Salmonella Typhimurium* and hepatitis A in spinach, lettuce, tomatoes, lettuce, and carrots (CDC, 2006; Everis, 2004). Consumption of fresh vegetables has increased mainly because of increased awareness of the benefits of a healthy diet (Anon, 2005). This has led to increased consumer demand for improved quality and safe vegetables especially in minimally processed and ready‐to‐eat fruit vegetables (Everis, 2004).

Cooking is a well‐known procedure in vegetable processing. The temperature is always at 100°C for a period of time. The time ranges between 1 and 10 min, depending on product requirements. Cooking has several advantages. It removes foreign materials, improves product color, inactivates enzymes that would otherwise cause undesirable changes in vegetables, and improves the texture (Nguyen‐the & Carlin, 1994). In industries, heat treatment such as blanching aid in filling of cans and improves the microbial quality of vegetables.

Abakaliki, like other cities in the developing countries, is expanding tremendously, without proper planning of utilities, safety, and regard to environment, making resident dump waste into rivers and on land. These wastes may contain heavy metals among other contaminants (Yebpella et al., 2011). Food safety is a major public health concern worldwide. During the last decades, the increasing demand on food safety has stimulated research regarding the risk associated with consumption of food stuffs contaminated with pathogenic microorganisms as well as potentially toxic heavy metals. Thus, the purpose of this study was to assess the heavy metal and microbial safety of raw and cooked pumpkin and *Amaranthus viridis* leaves grown in Abakaliki metropolis.

## 2 MATERIALS AND METHOD

### 2.1 Samples

Pumpkin Leaf and *A. viridis* leaves.

### 2.2 Sample collection

The samples (500 g) each were collected from vegetable farms in three different locations: Aziyokwu [V_1_], Udemuzue [V_2_], and Nna street [V_3_]) in Abakaliki metropolis. The samples were taken immediately to the Department of Food Science and Technology, Ebonyi State University for analysis.

### 2.3 Sample preparation

Each sample was divided into two: one from each location was cooked separately for 5 min at 100°C using gas stove, and the other was left raw. The raw and the cooked samples were analyzed for heavy metals and microbial quality.

### 2.4 Heavy metal analysis

#### 2.4.1 Methods for the heavy metal analysis

Heavy metal analysis was carried out using Varian (USA) AAS (atomic absorption spectrophotometer) according to the method of APHA 1995 (American Public Health Association) at spring board laboratory service Awka, Anambra State.

The following heavy metals were determined lead (Pb), cadmium (Cd), mercury (Hg), arsenic (As), and chromium (Cr).

#### 2.4.2 Preparation of reference solution and the heavy metal determination

A series of standard metal solutions in the optimum concentration ranges were prepared, and the reference solution was prepared by diluting the single stock element solutions with distilled water containing 1.5 ml concentrated nitric acid/liter. A calibration blank was prepared using all the reagents except for the metal stock solution. The samples were introduced into the AAS machine, and the quantity of each heavy metal was determined based on the working principle of AAS. Calibration curve for each metal was prepared by plotting the absorbance standards versus their concentrations. The curves were printed from the AAS machine.

### 2.5 Total viable bacterial and fungal count

Tenfold serial dilution was used for both fungal and bacterial counts. All media used (nutrient agar and Sabouraud dextrose agar [SDA]) were prepared according to manufacturer's instruction (BIOTECH India) and autoclave for 15 min at 121°C and 15 psi. The prepared media were allowed to cool to about 40°C in a water bath and then poured into sterile petri dishes containing 1 ml aliquot of the appropriate dilutions (peptone water as diluents) prepared from the samples. The total bacterial and fungal count was made using 10‐fold serial dilution in which 1 g of ground vegetable was put in 10 ml of peptone water as diluents, and one ml of the liquid was transferred to 9 ml of peptone water. Tenfold serial dilution of each sample was performed until 10^9^ level of dilution was obtained. One milliliter of the 10^6^, 10^5^, and 10^4^ of the dilution was plated out on nutrient agar and SDA for the determination of microbial counts of the sample. The samples were incubated at laboratory temperature of 28–29°C for 48 and 72 hr for bacterial and fungal, respectively.

Total viable counts of bacteria and fungi cells were determined by enumerating the colony‐forming units (cfu/g) at the end of the incubation period using the following formula:

$$C=\frac{n}{\mathrm{Vd}}$$

where *C* = colony‐forming unit per gram (cfu/g); *N* = number of colonies; *D* = dilution blank factor; *V* = volume transferred to plate.

### 2.6 Bacterial and fungal identification

The bacterial and fungal colonies were subcultured on nutrient and SDA and sent to CABI for molecular identification.

### 2.7 Methods for molecular identification of isolates

Fungal samples were processed using ITS rDNA sequencing analysis using the FASTA algorithm with the Fungus database from EBI. Bacteria samples were processed using partial 16S rDNA sequencing analysis. All procedures were validated and processing undertaken in accordance with CABI's in‐house methods as documented in TPs 61‐68 and TP70 for bacteria and TPs 72‐80 for filamentous fungi. All original samples were subjected to a purity check. Molecular assays were carried out on each sample using nucleic acid as a template. A proprietary formulation [microLYSIS^®^‐PLUS (MLP), Microzone, UK)] was subjected to the rapid heating and cooling of a thermal cycler, to lyse cells and release deoxyribonucleic acid (DNA). Following DNA extraction, polymerase chain reaction (PCR) was employed to amplify copies of the rDNA in vitro. The quality of the PCR product was assessed by undertaking gel electrophoresis. Polymerase chain reaction purification step was carried out to remove unutilized dNTPs, primers, polymerase, and other PCR mixture compounds and obtain a highly purified DNA template for sequencing. This procedure also allowed concentration of low‐yield amplicons. Sequencing reactions were undertaken using BigDye^®^ Terminator v3.1 kit from Applied Biosystems (Life Technologies, UK) which utilizes fluorescent labeling of the chain terminator ddNTPs, to permit sequencing. Removal of excess unincorporated dye terminators was carried out to ensure a problem‐free electrophoresis of fluorescently labeled sequencing reaction products on the capillary array AB 3130 Genetic Analyzer (DS1). DyeEx^™^ 2.0 (Qiagen, UK) modules containing prehydrated gel‐filtration resin were optimized for cleanup of sequencing reactions containing BigDye^®^ terminators. Dye removal was followed by suspension of the purified products in highly deionized formamide Hi‐Di^™^ (Life Technologies) to prevent rapid sample evaporation and secondary structure formation.

Samples were loaded onto the AB 3130 Genetic Analyzer and sequencing undertaken to determine the order of the nucleotide bases, adenine, guanine, cytosine, and thymine in the DNA oligonucleotide. Following sequencing, identifications were undertaken by comparing the sequence obtained with those available in European Molecular Biology Laboratory (EMBL) via the European Bioinformatics Institute (EBI).

## 3 RESULTS AND DISCUSSION

### 3.1 Heavy metal analysis of the raw and cooked pumpkin and *Amaranthus viridis* leaves

The results of the heavy metal analysis of the cooked and raw pumpkin and *A. viridis* leaves are presented in Tables 1 and 2.

**Table 1:** Heavy metals analysis of uncooked and cooked pumpkin leaves

Heavy metals (mg/kg)	V_1_	V_1_C	V_2_	V_2_C	V_3_	V_3_C
Pb	10.5 ± 0.02^a^	6.8 ± 0.01^b^	7.8 ± 0.13^c^	4.3 ± 0.03^d^	9.3 ± 0.53^e^	6.2 ± 0.05^f^
As	5.3 ± 0.02^a^	3.3 ± 0.02^b^	4.4 ± 0.02^c^	2.8 ± 0.02^d^	7.5 ± 0.02^e^	5.1 ± 0.02^f^
Cr	0.9 ± 0.01^a^	0.6 ± 0.05^b^	0.3 ± 0.02^c^	0.1 ± 0.02^d^	0.0	0.00
Cd	0.6 ± 0.02^a^	0.3 ± 0.01^b^	0.1 ± 0.01^c^	0.1 ± 0.00^c^	0.1 ± 0.01^e^	0.1 ± 0.01^f^
Hg	13.1 ± 0.01^a^	9.5 ± 0.01^b^	5.7 ± 0.01^c^	2.9 ± 0.02^d^	7.8 ± 0.01^e^	4.0 ± 0.0^f^

*Notes*.

Values are standard deviation of replicate samples.

Values with the same superscripts along the row are not statistically different (*p* > 0.05).

V_1_, V_1_C, V_2_, V_2_C, V_3_, V_3_C are pumpkin from locations 1, 2, 3 before and after cooking, respectively.

**Table 2:** Heavy metal analysis of cooked and uncooked *Amaranthus viridis*

Heavy metals (mg/kg)	V_1_	V_1_C	V_2_	V_2_C	V_3_	V_3_C
Pb	12.0 ± 40.02^a^	8.4 ± 0.01^b^	8.2 ± 0.02^c^	5.3 ± 0.02^d^	9.8 ± 0.01^e^	6.6 ± 0.02^f^
As	7.5 ± 0.02^a^	3.9 ± 0.03^b^	6.4 ± 0.02^c^	4.4 ± 0.01^d^	8.5 ± 0.02^e^	6.1 ± 0.01^f^
Cr	0.1 ± 0.001^a^	0.1 ± 0.00^b^	0.1 ± 0.00^c^	0.1 ± 0.00^c^	0.0	0.0
Cd	0.9 ± 0.03^a^	0.5 ± 0.01^b^	0.8 ± 0.01^c^	0.5 ± 0.01^d^	0.8 ± 0.01^e^	0.5 ± 0.01^f^
Hg	14.0 ± 0.02^a^	11.4 ± 0.01^b^	8.9 ± 0.03^c^	5.9 ± 0.02^d^	3.8 ± 0.02^e^	0.9 ± 0.01^f^

*Notes*.

Values are standard deviation of replicate samples.

Values with the same superscripts along the row are not statistically different (*p *> 0.05).

V_1_, V_1_C, V_2_, V_2_C, V_3_, V_3_C are pumpkin from locations 1, 2, 3 before and after cooking, respectively.

#### 3.1.1 Lead

Lead is a toxic and widely distributed heavy metal. It can be harmful to both plants and animals. Although plants usually show ability to accumulate large amounts of lead without visible changes in their appearance or yield. In many plants, including leafy vegetables, lead accumulation can exceed several hundred times the threshold of maximum level permissible for human consumption (Choi, 2011). Thus, in humans, significant source of exposure is often from contaminated food and water (EFSA, 2009). The finding of the current study showed that the raw vegetables were contaminated with Pb and the vegetables still retain significant levels of the heavy metal after cooking (Tables 1 and 2). The level of lead in the current study ranged from 4.27 to 10.52 mg/kg for pumpkin and 6.55 to 12.04 mg/kg for *A. viridis* leaves. These levels of lead contamination portent a significant health risk for the consumers of these leafy vegetables (EC, 2004). Lead exposure through contaminated food and water has been directly linked with the risk of developmental neurotoxicity in young children, cardiovascular effects, and nephrotoxicity in adults. According to Food Safety Authority of Ireland, short‐term exposure to high levels of lead can cause brain damage, paralysis (lead palsy), anemia, and gastrointestinal symptoms. Long‐term exposure can cause damage to the kidneys, reproductive, and immune systems in addition to effects on the nervous system (FSAI, 2009; Itanna, 2003). The lead contents of the vegetables in this study are higher when compared to the FAO/WHO safe limit of 0.3 mg/kg (JECFA, 2003). According to European Food Safety Authority, the maximum acceptable limit of lead in vegetables is 0.1 mg/kg (EFSA, 2004), while maximum acceptable limit for lead in food according to FSANZ is 0.1 mg/kg (FSANZ, 2011). The vegetables might have absorbed this metal from the soil as well as the irrigation water. The irrigation waters are from shallow wells (dogged solely for irrigation purposes) contaminated with industrial and domestic sewage channeled from household and industrial site within the metropolis. The levels of the lead contamination were significantly higher in the raw vegetables compared with the cooked vegetables in the current study though still not within international acceptable limit. Thus cooking was not able to remove the incidence of lead contamination in the vegetables. The decrease in the lead concentration in the cooked vegetables could be as a result of osmotic leaching of the heavy metal from the vegetables to the cooking water. The high levels of lead in these vegetables (fresh and cooked) present a significant health risk for the consumers of these vegetables.

#### 3.1.2 Arsenic

Arsenic exists both in organic and in inorganic forms and also in different valence states. Arsenic has been classified by the International Agency for Research into Cancer (IARC) as a human carcinogen on the basis of increased incidence of cancers at several sites in people exposed to arsenic at work environment or through their diet (FSAI, 2009). It is also more acutely toxic than other metallic compounds and was used in earlier times as a rodenticide. Continuous low‐level exposure to arsenic can cause skin, vascular, and nervous system disorders (Wilson, 2011; Yebpella et al., 2011). Arsenic enters the food chain through contaminated water and soil. The finding of the current study indicates that the arsenic contents of the raw and cooked pumpkin and *A. viridis* leaves were above international acceptable limit (Tables 1 and 2). According to WHO/FAO, the maximum acceptable limit of arsenic in food is 0.1 mg/kg (JECFA, 2003). The arsenic levels of cooked and raw pumpkin and *A. viridis* leaves were statistically different (*p* > 0.05). It was also relatively more abundant in location V_3_ (Nna St.) and lowest in location V_2_ (Udemuezue). Consumption of these vegetables would certainly result in dangerous health consequences including kidney and liver damage, gastrointestinal effects, and damage of DNA (FSAI, 2009). The result of this research is similar to the finding of Itanna (2003) who reported high concentrations of arsenic in different vegetables studied. According to the report, the concentrations of arsenic in the vegetables were high enough to cause clinical damage both to animals and to humans consuming the metal‐rich vegetables.

#### 3.1.3 Chromium

Chromium has two common oxidation states, Cr (iii and iv), and has been reported to exert toxic effect on biological systems (Sobukola et al., 2010). The vegetable samples were also found to be contaminated with chromium (Tables 1 and 2). However, the concentrations of the Cr metal on the raw and the cooked vegetables were within tolerable limit. The maximum acceptable limit of chromium in vegetables is 1 ppm (EC, 2008; Choi, 2011), while the result of this study showed that all the samples analyzed were below 1 ppm; however, it is important to note there need for continuous monitoring of the metal in the commonly consumed vegetables. Chromium content of the two vegetables from the different locations differed significantly (*p* > 0.05) and cooking had a significant effect in reducing the chromium level. The maximum permissible limit of chromium for plant is 1.30 mg/kg recommended by World Health Organization (WHO) (Wilson, 2011). The standard for irrigation water approved by National Environmental Quality standards (NEQS) for chromium is 1.0 Ng/mil. Mohammed and Folorunsho (2015) reported a chromium level of 0.058 to 2.80 mg/kg in *Amaranthus retroflexus*. This finding is similar the finding of Muchuweti et al. (2005) in which they disclosed that the concentrations of chromium in some of the vegetables and fruit samples studied were within tolerable limit but need to be continuously monitored.

#### 3.1.4 Cadmium

The finding of this research revealed that the concentrations of cadmium were higher than the maximum acceptable limits of cadmium in food which is 0.1 mg/kg in vegetables and cereals according to International Standards for Heavy Metals in Food (JECFA, 2003). According to European Union regulation, the maximum acceptable limit of cadmium in leafy vegetables is 0.02 mg/kg (EC, 2004). This result is similar to the work of other researchers in which they revealed that high concentrations (above acceptable international standards) of Cd content in fruits and vegetables grown in soil and/or irrigation water contaminated with the metal (FSAI, 2009; Suruchi & Jilani, 2011). Preventing the incidence of Cd absorption and poisoning in human and animals from source including vegetables is clinically important according to European Parliament and Council Regulation (EC, 2002). In some, many different organ dysfunctions have been linked to Cd in humans such as renal dysfunctions and bone demineralization. The International Agency for Research on Cancer has also classified Cd as a group 1 human carcinogen (EFSA, 2009, 2004). Cooking also had a significant effect on the cadmium level of the vegetables from the different locations but was unable to reduce it to the safe limit of 0.2 mg/kg as provided by the Food Safety Authority Ireland (FSAI, 2009). The principal toxic effect of cadmium is its toxicity to the kidney but has also been associated with lung damage (including induction of lung tumors) and skeletal changes in exposed population (Muchuweti et al., 2005; Choi, 2011). This goes to show that consumers of these vegetables are exposed to high health risk and are bound to have many organ failures in the future if something is not done urgently to address this potentially health/environmental disaster.

#### 3.1.5 Mercury

The levels of mercury contamination of the raw and cooked vegetables were above tolerable limits. However, cooking significantly (*p* > 0.05) reduced the mercury levels compared to the raw pumpkin and *A. viridis* but was still above acceptable limit (Tables 1 and 2). According to WHO, the maximum acceptable limit of mercury is 1 μg/kg. It has been established that environmental contaminations of mercury can both be from natural sources and from anthropogenic emissions such as industrial activities and mining. According to European Food Safety Association (EFSA) and US food and Drug Administration (FDA), the minimum acceptable limit of mercury in food is 0.5 mg/kg and 1 μg/L for water (FSAI, 2009; EFSA 2009). The findings of the current study showed that the raw pumpkin and *A. viridis* leaves have high mercury contaminations and are of serious concern considering that these vegetables are consumed in high volume within the metropolis. Excessive exposure to mercury through contaminated foods and water has been associated with a wide spectrum of adverse health effects including damage to the central nervous system (neurotoxicity) and the kidney (Sobukola et al., 2010; EC, 2004). The levels of the metal contamination among the three locations between pumpkin and *A. viridis* leaves were significantly (*p* > 0.05) different. This further showed that there is an urgent need not only to continuously monitor the extent of heavy metal contaminations of the vegetables but also for government to take urgent steps to stop or curtail sell the heavy metal contaminated vegetables in Abakaliki metropolis.

### 3.2 Result of the total viable count/molecular identification of the fungal and bacterial isolates from the raw and cooked pumpkin and *Amaranthus viridis* leaves

The results of the current research showed that both pumpkin and *A. viridis* leaves were heavily contaminated with microorganisms (Tables 3 and 4). The levels of the microbial (bacteria and fungi) contaminations of the vegetables were beyond maximum acceptable limits according to international commission on microbiological specification of food. According to this agency, the maximum acceptable limit of bacteria count in food products is 10^3^ cfu/ml (ICMSF, 1995). In the current study, the numbers of the microbial count were above this limit, thus present potential hazard as these vegetables are often consumed raw. The number of documented outbreaks of human infections associated with the consumption of raw fruits, vegetables, and unpasteurized fruit juices has increased in recent years according to the Centers for Disease Control and Prevention, in the United States (CDC, 2006). The reasons for this increase in produce‐related human infections were proposed to include changes in dietary habits, including a higher per capita consumption of fresh or minimally processed fruits and vegetables (Buck et al., 2003 and Abadias et al., 2008).

**Table 3:** Microbial count of pumpkin leaves

Microbial count (cfu/g)	V_1_	V_1_C	V_2_	V_2_C	V_3_	V^3^C
Bacterial count	4.8 × 10^8^ ± 0.01^a^	1.1 × 10^6^ ± 0.01^b^	3.8 × 10^8^ ± 0.06^c^	0.00	5 × 10^8^ ± 0.1^d^	0.00
Fungi count	8.2 × 10^4^ ± 0.1^a^	0.00	1.3 × 10^6^ ± 0.01^b^	0.00	2.8 × 10^6^ ± 0.05^d^	0.00

*Notes*.

Values are standard deviation of replicate samples.

Values with the same superscripts along the row are not statistically different (*p* > 0.05).

V_1_, V_1_C, V_2_, V_2_C, V_3_, V_3_C are pumpkin from locations 1, 2, 3 before and after cooking, respectively.

**Table 4:** Microbial Count of the *Amarantus viridis*

Microbial count (cfu/g)	V_1_	V_1_C	V_2_	V_2_C	V_3_	V^3^C
Bacterial count	3.1 × 10^1^ ± 0.1^a^	1.7 × 10^6^ ± 0.07^b^	5.4 × 10^6^ ± 0.05^c^	0.00	7 × 10^7^ ± 0.15^d^	0.00
Fungi count	1.7 × 10^3^ ± 0.1^a^	0.00	2 × 10^5^ ± 0.15^b^	0.00	5 × 10^6^ ± 0.15^d^	0.00

*Notes*.

Values are standard deviation of replicate samples.

Values with the same superscripts along the row are not statistically different (*p *> 0.05).

V_1_, V_1_C, V_2_, V_2_C, V_3_, V_3_C are pumpkin from locations 1, 2, 3 before and after cooking, respectively.

Microbial contamination of fruits and vegetables is directly linked to the hygienic practices during their production, harvesting, postharvest handling, processing, and distribution of the product (Heaton & Jones, 2008). Soil and irrigation water especially those contaminated with industrial and domestic wastes have also been established to be a common source of microbial contamination of leafy vegetables (Mapanda et al., 2005). This agrees with finding of this study as the high microbial load on the vegetables could be from both the irrigation waters and/or the soil. Cooking completely eliminated the contaminating organisms except for vegetables from location one which retained some microbial cells after cooking (Francis et al., 1999). The effectiveness of cooking as a processing method in sufficiently reducing/eliminating the microbial contaminant explains why incidences of outbreak foodborne illness have always been associated with raw or semiprocessed vegetables not properly cooked vegetables. This implies that majority of the contaminating microbial cells are possibly enteric organisms while the vegetables from location one might have had mixed contamination with enteric as well as spore‐forming organisms. Enteric pathogens such as *E. coli*,* Enterobacter* spp., *Klebsiella* spp., *and Bacillus* spp. have all often been isolated from fresh leafy and salad vegetables (Denis, Zhang, Leroux, Trudel, & Bietlot, 2016; Szabo, Scurrah, & Burrows, 2000). According to (Lennox & Efiuvwevwere, 2012), pumpkin leaves are vulnerable to microbial contamination from the soil and irrigation water.

The result of the molecular identification of the most dominant bacteria contaminant in the samples further confirms the potential danger associated with these vegetables as the bacteria isolate shows top matches (97–98%) to the species *E. coli* and *Escherichia fergusonii* (504743*) and Klebsiella pneumonia* (504744b). Thus, the isolates were identified as *E. coli* and *E. fergusonii* and *K. pneumonia* (CABI Identification Services UK). *Escherichia coli* and *E. fergusonii* belong to the family Enterobacteriaceae and have been established to be distributed worldwide and can be found in soil, water, plants, food products, animals, and human. Some members of this species (including enteropathogenic *E. coli*) are pathogenic to human and have been categorized as hazard group 2 or 3 organisms by Advisory Committee on Dangerous Pathogens (ACDP) (UK). Enteric pathogens such as *E. coli* O157:H7 and *Salmonella* spp. infection has been associated with carrots, lettuce, sprouts, and apple juice, and leafy vegetables (Buck et al., 2003). The sequence obtained from the fungi isolated from the vegetable samples showed top matches at 100% identity to multiple sequences from strains of *Aspergillus flavus* (504738). This is a common species especially in the tropics, with brown to olive colonies and large thick‐welled roughened conidia. It is frequently isolated from soil, seeds, and other plant‐based substrata and is known to produce the mycotoxin aflatoxins. It is assigned by ACDP (UK) to hazard group 2 thus substrate containing this organism is designated as potentially dangerous and should be discarded according to ACDP (UK). Fungi species such as *Penicillium expansum has been isolated from raw* on raw fruits and vegetables where they can result in the formation of biofilms by spoilage and nonspoilage microorganisms. These biofilms provide a protective environment for pathogens and reduce the effectiveness of sanitizers and other simple processing technique such as washing and blanching that would have otherwise reduced the microbial load of the vegetables (Nguyen‐the & Carlin, 1994; Burnett & Beuchat, 2001 and Buck et al., 2003) (Table 5).

**Table 5:** Comparison of heavy metals concentrations in the pumpkin and *Amaranthus* leaves to WHO/EU permissible limits

Heavy metals (mg/kg)	P1	A1	P2	A2	P2	A3	WHO/EU limit
Pb	10.5	12.0	7.8	8.2	9.3	9.8	0.1
As	5.3	7.5	4.4	6.4	7.5	8.5	0.1
Cr	0.9	0.1	0.3	0.1	0.0	0.0	1.3
Cd	0.6	0.9	0.1	0.8	0.1	0.8	0.02
Hg	13.1	14.0	5.7	8.9	7.8	3.8	1.0

*Notes*.

P1, P2, P3 is the pumpkin leaves from the three locations, while A1, A2, A3 is the *Amaranthus viridis* leaves from the three locations.

The finding of the current study correlates FAO/WHO report on microbiological hazard from foods which states that leafy green vegetables currently pose the greatest concern in terms of microbiological hazards (CAC, 2003). The sources of this microbial contamination could be from domestic and industrial sewage that often channeled to the irrigation water. According to U.S National Advisory Committee on Microbiological Criteria for Foods (NACMCF), potential preharvest sources of contamination of vegetables include soil, feces, irrigation water, water used to apply fungicides and insecticides, dust, insects, inadequately composted manure, wild and domestic animals, and human handling (NACMCF, 1999). Dumping/channeling of domestic and industrial waste to irrigation site has been observed to be common practices among resident in Abakaliki metropolis. Lack of freshwater for irrigation purposes have forced growers to utilize any type of available water, including wastewater from shallow wells, contaminated with domestic and industrial sewage. The finding of this study calls for an urgent intervention by government to avoid possible outbreak of food poisoning from heavy metal and microbial contaminated pumpkin and *A. viridis* leaves.

## 4 CONCLUSION/PRACTICAL IMPLICATION

The finding of this research revealed that the pumpkin and *A. viridis* leaves grown in Abakaliki metropolis contain high concentrations of potentially toxic heavy metals. The microbial content of the vegetables was also above safe limit and the nature of the isolated microorganisms is potentially dangerous. Therefore, there is an urgent need for an urgent action plan that will properly identify the source of these contaminations and ensure that proper solutions are urgently provided to prevent possible outbreak of food poisoning.

## CONFLICT OF INTEREST

This is to declare that all the authors agreed to publish the article in the Journal of Food Science and Nutrition; hence, there is no conflict of interest among authors. The research was conducted within the guidelines of Ebonyi State University Research Council and the guidelines of Nigerian Research Council. There is no ethical problem in the research.
